# Variant- and vaccination-specific alternative splicing profiles in SARS-CoV-2 infections

**DOI:** 10.1016/j.isci.2024.109177

**Published:** 2024-02-08

**Authors:** Sung-Gwon Lee, Priscilla A. Furth, Lothar Hennighausen, Hye Kyung Lee

**Affiliations:** 1 Section of Genetics and Physiology, Laboratory of Molecular and Cellular Biology, National Institute of Diabetes and Digestive and Kidney Diseases (NIDDK), National Institutes of Health (NIH), Bethesda, MD, USA

**Keywords:** Immunology, Molecular biology, Virology

## Abstract

The COVID-19 pandemic, driven by the SARS-CoV-2 virus and its variants, highlights the important role of understanding host-viral molecular interactions influencing infection outcomes. Alternative splicing post-infection can impact both host responses and viral replication. We analyzed RNA splicing patterns in immune cells across various SARS-CoV-2 variants, considering immunization status. Using a dataset of 190 RNA-seq samples from our prior studies, we observed a substantial deactivation of alternative splicing and RNA splicing-related genes in COVID-19 patients. The alterations varied significantly depending on the infecting variant and immunization history. Notably, Alpha or Beta-infected patients differed from controls, while Omicron-infected patients displayed a splicing profile closer to controls. Particularly, vaccinated Omicron-infected individuals showed a distinct dynamic in alternative splicing patterns not widely shared among other groups. Our findings underscore the intricate interplay between SARS-CoV-2 variants, vaccination-induced immunity, and alternative splicing, emphasizing the need for further investigations to deepen understanding and guide therapeutic development.

## Highlights

• SARS-CoV-2 variants impact host alternative splicing and infection outcomes
• Different SARS-CoV-2 variants induce distinct alterations in RNA splicing
• Vaccination status influences alternative splicing patterns in COVID-19 patients
• Alternative splicing machinery is abnormally regulated in COVID-19 patients

## Graphical abstract

**Figure:**
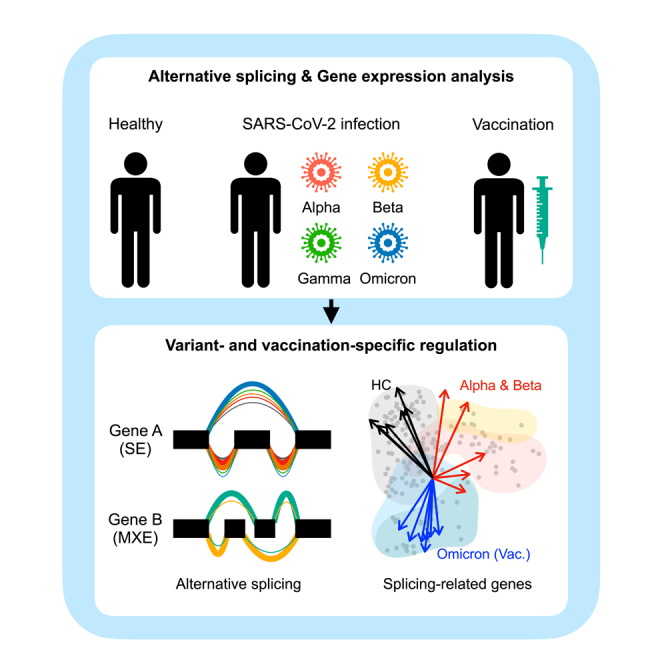

## Introduction

The emergence of the coronavirus SARS-CoV-2, the causative agent of COVID-19, has precipitated a global public health crisis.^1^^,^^2^^,^^3^ The SARS-CoV-2 infection manifests a broad spectrum of symptoms ranging from mild respiratory discomfort to severe acute respiratory syndrome, with potential long-term sequelae.^4^^,^^5^ The quest to unravel the molecular mechanisms of SARS-CoV-2 infection and propagation has burgeoned into genomic, transcriptomic, and proteomic dimensions.^6^^,^^7^^,^^8^

Alternative splicing is a cellular process that increases the number of mRNA isoforms that can augment proteomic diversity and function as well as compromising protein expression through loss of open reading frames and switching from coding to non-coding transcripts.^9^^,^^10^^,^^11^ It has been implicated in various diseases including viral infections, where it is poised to play a crucial role in modulating host immune responses and viral replication mechanisms.^12^^,^^13^^,^^14^^,^^15^ This is also true of SARS-CoV-2 infection.^11^^,^^15^^,^^16^^,^^17^^,^^18^ Specific alternative spliced transcripts present with SARS-CoV-2 infection can lead to reduced antiviral immunity. Examples of previously identified alternatively spliced genes include CD74 and LRRFIP115, and *OAS1.*^18^ A specific SARS-CoV-2 protein, NSP16, has been shown to bind to the U1 and U2 splicing RNAs.^16^ In short, alternative splicing is an aspect of molecular biology that can contribute to the pathophysiology of SARS-CoV-2 infection. But, despite the research directed toward understanding feature of SARS-CoV-2 variants, alteration of immune response induced by infection and vaccination, and genome-wide transcriptome alterations,^19^^,^^20^^,^^21^^,^^22^^,^^23^^,^^24^ a conspicuous research gap persists concerning alternative splicing profiles and the splicing machinery that can be affected by variants and vaccination statuses.

Here, we analyzed 190 RNA-seq datasets from five COVID-19 cohorts across four variants infected patients (Alpha, Beta, Gamma, and Omicron) and healthy controls to identify their transcriptome profiles including alternative splicing and gene expression. We also investigated variant- and vaccination-specific transcriptional regulations. From these analyses, we discovered the dysregulation of alternative splicing and its machinery genes in COVID-19 patients, with specific regulation patterns associated with variants and vaccination statuses. This examination allowed us to not only explore the intricate transcriptional landscape underpinning the infection dynamics of different SARS-CoV-2 variants but also the potential modulatory impact of vaccination on host transcriptome.

## Results

### Landscape of splicing across COVID-19 patients infected with four SARS-CoV-2 variants reveals an aberrant global alternative splicing pattern

**Figure 1:**
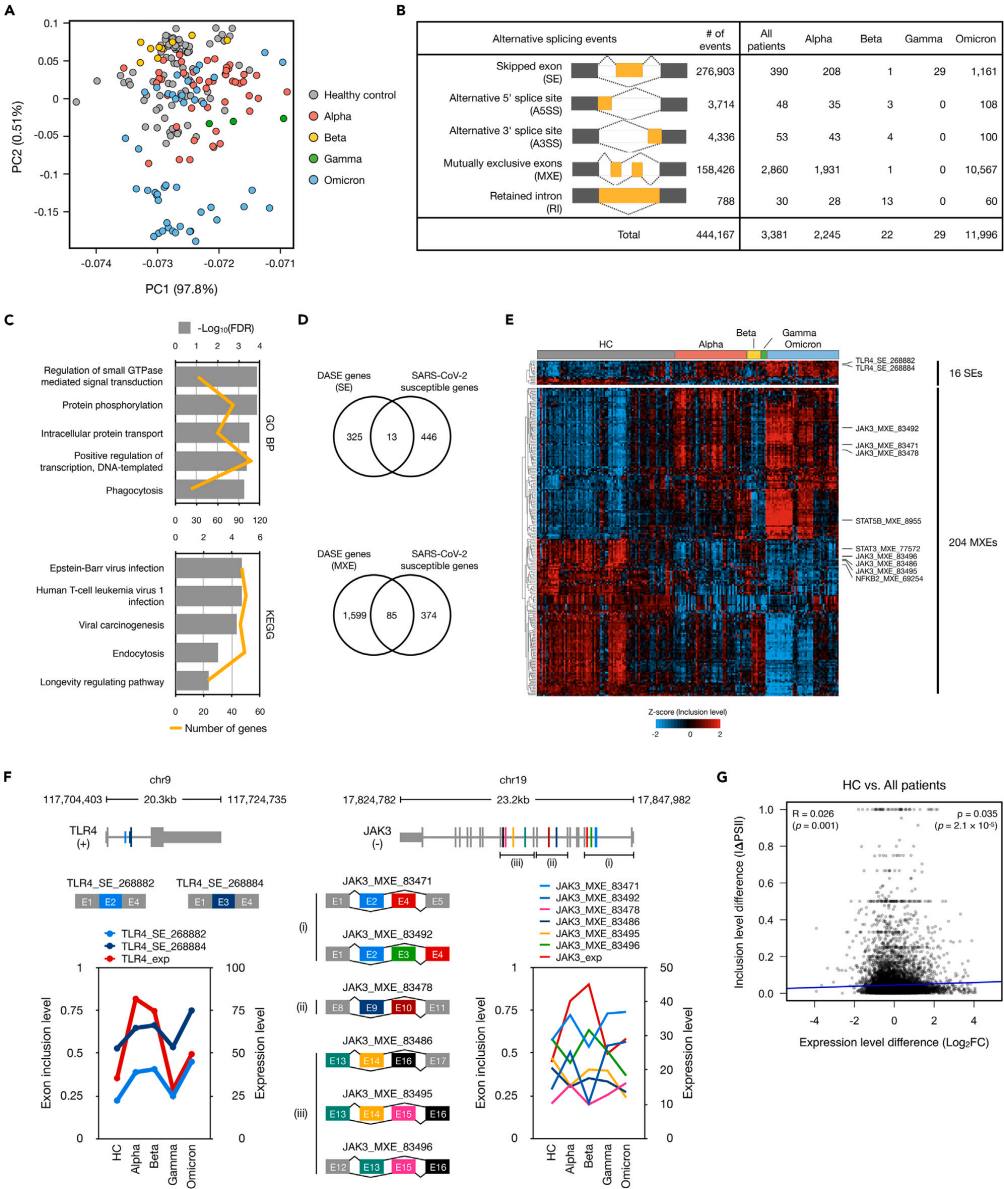
SARS-CoV-2 infection leads to aberrant global alternative splicing (A) Principal component analysis (PCA) plot of exon inclusion level from 190 RNA-seq dataset. (B) Number of differential alternative splicing events (DASEs) of five alternative splicing types. The DASEs were defined as following criteria: absolute PSI differences value >0.1 and corrected p value <0.05. (C) Results of the top 5 terms of GO biological process and KEGG pathways enriched with the 1,928 genes from 3,381 DASEs (HC vs. All patients). (D) Venn diagrams displaying the gene overlap between DASEs in the SE and MXE categories, respectively, and the set of SARS-CoV-2 susceptible genes. (E) Heatmap of differentially alternative spliced SARS-CoV-2 susceptible genes between HC and COVID-19 patients. *Z* score indicates relative exon inclusion levels. Hierarchical clustering of DASEs was performed with a Euclidean distance matrix of relative exon inclusion levels. (F) Significant differential spliced events of *TLR4* (chr9:117,704,403-117,724,735) and *JAK3* (chr19:17,824,782-17,847,982) showing two skipped exon (SE) events and six mutually exclusive exon (MXE) events, respectively. Exon inclusion levels represent the usage of spliced exons in the case of SE events, while in the context of MXE events, they indicate the ratio of the second mutually exclusive exon within each event. The expression level refers to the overall gene expression level. (G) Correlation analysis of expression differences between the inclusion level differences of total genes.

To investigate the impacts of SARS-CoV-2 variants on alternative splicing of cellular RNAs, we analyzed 190 RNA-seq data from buffy coats of COVID-19 patients infected with four different variants (Alpha, Beta, Gamma, and Omicron), as well as healthy controls (HC) (Table S1).^20^^,^^21^^,^^25^^,^^26^^,^^27^ We specifically focused on data from patients within one week of infection to investigate transcriptional changes at an early stage. In total, approximately 40.3 billion reads were mapped to the human genome, achieving an average alignment rate of 95.6%. Using the rMATs,^28^ we identified 444,167 alternative splicing events and estimated their exon inclusion levels. Principal component analysis (PCA) showed intermingled profiles of most samples except for Omicron-infected patients (Figure 1A). A total of 3,381 differential alternative splicing events (DASEs) spanning five distinct alternative splicing categories were identified in COVID-19 patients compared to HC (Figure 1B). We observed 2,245 DASEs in Alpha-infected patients, while dozens of DASEs were found in Beta- and Gamma-infected patients. Interestingly, 11,996 DASEs were specifically identified in Omicron-infected patients. This finding suggested that the alternative splicing is globally modified in COVID-19 patients.

The 1,928 genes from 3,381 DASEs were significantly enriched in GO terms of general cellular functions including regulation of small GTPase mediated signal transduction (GO:0051056; FDR = 1.4 ✕ 10^−4^), intra cellular protein transport (GO:0006886; FDR = 3.3 ✕ 10^−4^) and transcription (GO:0045893; FDR = 4.1 ✕ 10^−4^) (Figure 1C). Moreover, they are mainly involved in virus infection pathways including Epstein-Barr virus (hsa05169; FDR = 2.0 ✕ 10^−5^) and human T cell leukemia virus 1 (hsa05166; FDR = 2.0 ✕ 10^−5^). Indeed, these findings suggest that the genes exhibiting altered alternative splicing patterns during SARS-CoV-2 infection play a pivotal role in diverse cellular functions. Moreover, their pronounced involvement in virus infection pathways underscores their significance in shaping the observed alternative splicing patterns.

We compared the predominant alternative splicing events in our results, which comprise approximately 96% of the DASEs, specifically skipped exon (SE) and mutually exclusive exons (MXE) DASEs, with genes associated with susceptibility to viral infections, including SARS-CoV-2 and other viral infections. Thirteen genes (SE) and 85 genes (MXE) that exhibited associations with viral susceptibility were identified (Figure 1D). Those genes included 16 SEs and 204 MXEs, showing dynamic splicing alterations observed in COVID-19 patients (Figure 1E). Among DASEs, we found alternative exon usages of genes related to Janus kinase (JAK) signaling pathway and Toll-like receptor 4 (TLR4) (Figure 1F). Specifically, exon 2 and 3 of *TLR4* displayed higher inclusion levels in COVID-19 patients. Notably, these inclusion patterns have been previously reported in splice variants induced by lipopolysaccharide (LPS) treatment.^29^^,^^30^ The JAK signaling pathway plays a crucial role in virus infections, including those involving SARS-CoV-2.^31^ In our results, *JAK3* exhibited six novel MXE events spanning ten exons, indicating a wide range of alternative variants being generated. This underscores the complexity of alternative splicing of *TLR4* and *JAK3* and its potential significance in the context of viral infections.

We compared the transcript level difference and the absolute mean inclusion level difference between HC and COVID-19 patients to examine the relationship between relative gene expression levels and splicing rates in the context of SARS-CoV-2 infection. No correlation was seen between the levels of gene expression and alternative splicing rates (Figure 1G). This result is consistent with previous studies that found no correlations between changes in gene abundance and alternative splicing patterns during cell state transitions, development, or infection.^32^^,^^33^^,^^34^^,^^35^

### Dysregulated alternative splicing related genes in COVID-19 patients

**Figure 2:**
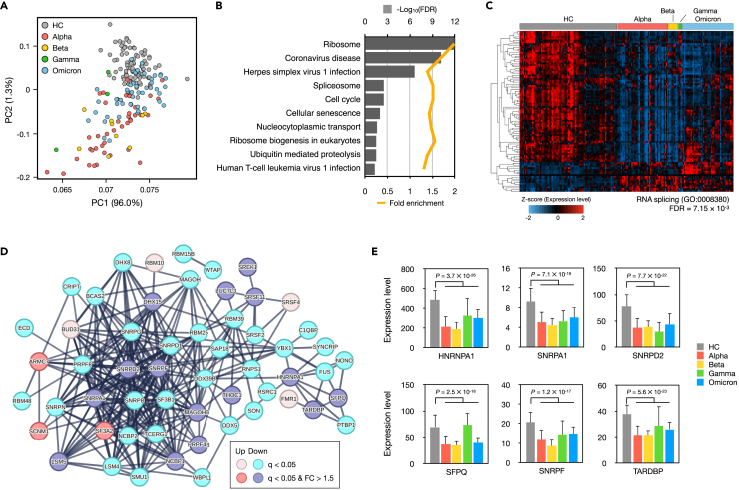
Dysregulated alternative splicing-related genes in COVID-19 patients (A) PCA plot of gene expression levels from 190 samples. (B) Results of the top 10 pathways enriched with the 7,529 DEGs. (C) Heatmap of 92 DEGs involved in RNA splicing (GO:0008380). *Z* score indicates relative gene expression levels. Hierarchical clustering of DEGs was performed with a Euclidean distance matrix of relative gene expression levels. (D) Protein-protein network of significant DEGs related to RNA splicing. The color of each gene indicates statistically significant and fold change to HC. (E) Gene expression levels of six hub genes which are depressed in COVID-19 patients. The error bar indicates standard deviation of the mean.

In our quest to identify genes that might influence alternative splicing changes in COVID-19, we examined global gene expression differences between healthy controls and COVID-19 patients. PCA results revealed pronounced gene expression differences primarily in Alpha and Beta-infected patients, while relatively minor differences were observed in Omicron-infected patients compared to healthy controls (Figures 2A and S1). We identified 7,529 genes that were differentially expressed (DEGs) in COVID-19 patients. Subsequent Gene Ontology (GO) analysis associated these DEGs with Coronavirus and Herpes simplex virus infections (Figure 2B). Interestingly, DEGs were notably enriched in spliceosome-related genes, suggesting dysregulation of spliceosome-related gene expression in COVID-19 patients. Independent Database for Annotation, Visualization and Integrated Discovery (DAVID) analysis also indicated significant enrichment of 92 DEGs in RNA splicing (GO:0008380), with the majority, 81.5% (75 out of 92 DEGs), being significantly down-regulated in COVID-19 patients (Figure 2C). Furthermore, we observed significantly reduced expression of hub genes such as *HNRNPA1*, *SNRPA1*, *SNRPD2*, *SFPQ*, *SNRPF*, and *TARDBP*, which interact intensively with other proteins in a protein-protein interaction network (Figures 2D and 2E). These findings collectively suggest that alternative splicing machinery is compromised in COVID-19 patients and is closely associated with abnormal global alternative splicing patterns.

### Different regulation of alternative splicing in T and B cell receptor signaling pathways in vaccinated and unvaccinated group of Omicron infected patients

**Figure 3:**
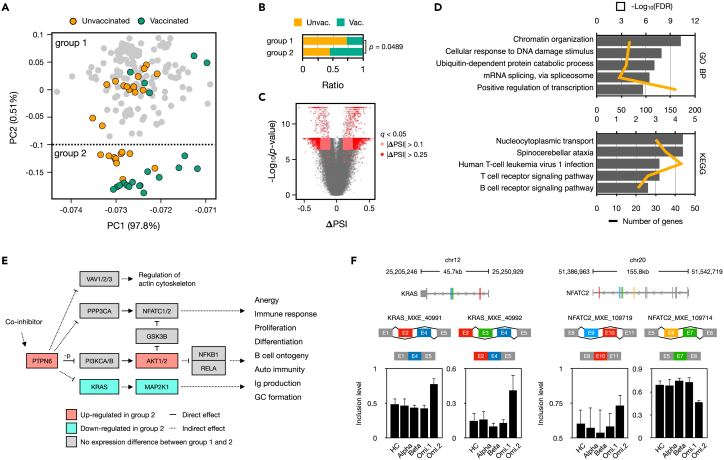
Differential regulation of alternative splicing in T and B cell receptor signaling genes among vaccinated and unvaccinated Omicron-infected patients (A) PCA plot of exon inclusion levels from 190 samples, as presented in Figure 1A. The samples are marked differentially based on the vaccination status of Omicron-infected patients. Group 1 and 2 represent clusters established through k-means clustering. (B) Vaccination status ratio of Omicron-infected patients within group 1 and group 2. A chi-squared test was conducted to confirm a significant difference in vaccination status proportions between the two groups. (C) Volcano plot showing the DASEs between Omicron groups. The x- and y axis indicate inclusion level difference (**Δ**PSI) and negative log_10_ transformed p value. The *q*-value indicated corrected p value. (D) Results of the top 5 terms of GO biological process and KEGG pathways enriched with the 1,789 genes from 2964 DASEs (Omi.group 1 vs. Omi.group 2). (E) A subset of shared pathways based on 16 common DASEs identified in the T cell receptor signaling pathway (hsa04660) and the B cell receptor signaling pathway (hsa04662). The coloration of each gene box signifies the expression difference between the two groups, with solid lines indicating a direct effect and dashed lines representing an indirect effect. (F) Significant differential spliced events of *KRAS* (chr12:25,205,246-25,250,929) and *NFATC2* (chr20:51,386,963-51,542,719) showing two MXE events, respectively. Inclusion levels represent the usage of spliced exons. The error bar indicates standard deviation of the mean.

We also observed distinct alternative splicing profiles among patients infected with the Omicron variant (Figure 1A). Through unsupervised clustering, we delineated two distinct groups within the entire patient cohort. Group 1 consisted of 163 samples (comprising HC, Alpha, Beta, and some Omicron cases), while group 2 comprised the remaining 27 Omicron-infected patients. Notably, we found that group 1 was enriched with unvaccinated individuals, while group 2 predominantly consisted of vaccinated patients (Figure 3A). The difference in vaccination status between these groups was statistically significant (Figure 3B).

We examined the alternative splicing events that changed between these two Omicron-infected patient groups and a total of 19,732 DASEs with the majority being of the MXE type were identified. With a more stringent criterion (an inclusion level difference >0.25), we identified 2,964 significant DASEs between Omicron group 1 and Omicron group 2 (Figure 3C). These DASEs were associated with 1,789 genes primarily related to general functions such as chromatin organization (GO:0006325; FDR = 5.4 ✕ 10^−11^), DNA damage response (GO:0006974; FDR = 1.2 ✕ 10^−8^), splicing (GO:0000398; FDR = 3.5 ✕ 10^−7^), transcription (GO:0045944; FDR = 2.1 ✕ 10^−6^), and were closely linked not only to virus infection (hsa05166; FDR = 6.2 ✕ 10^−4^) but also to the T (hsa04660; FDR = 6.2 ✕ 10^−4^) and B cell receptor signaling pathways (hsa04662; FDR = 2.5 ✕ 10^−3^) (Figure 3D).

Furthermore, we identified 16 downstream genes of the T and B cell receptor signaling pathways (Figure 3E). These genes, acting as intermediary genes, regulate immune processes including immune response, proliferation, and differentiation. Interestingly, most of these genes were regulated through splicing without exhibiting expression differences between the groups. Among them, *KRAS* was identified to have a higher inclusion of exon 4 in Omicron group 2 (Figure 3F). The isoform with exon 4 inclusion is reported as *KRAS4A* directly regulate glycolysis and apoptosis promotion and is predominantly expressed in endodermal organs.^36^^,^^37^^,^^38^ Another gene, *NFATC2*, showed an increased inclusion of exon 10 in Omicron group 2. The isoform with this inclusion has been reported to be ubiquitously expressed across various tissues,^39^ however, the decrease in exon 7 inclusion observed in Omicron group 2 represents a novel isoform which has not been reported previously. This implies that, depending on vaccination status, Omicron infection leads to differential regulation of alternative splicing in genes associated with both general cellular functions and immune cell signaling genes, reflecting broader changes in cellular function and immune response.

### Differential regulation of RNA splicing machinery genes across SARS-CoV-2 variants

**Figure 4:**
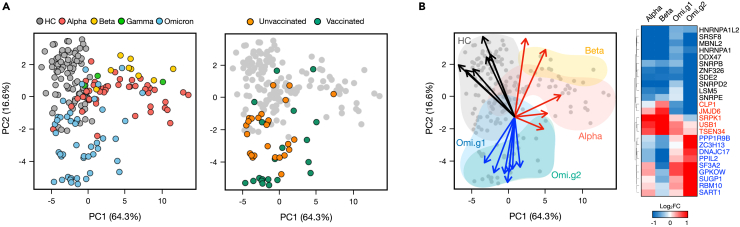
Patients infected with different variants exhibit differential regulation of splicing machinery genes (A) PCA plot of 304 genes related to RNA splicing across 190 samples. Each sample in HC and COVID-19 patients (left panel) and Omicron patients with vaccination status (right panel) is marked as dots. (B) Biplot results to identify representative genes with high contributions in each group. Blue arrows represent genes representing the Omicron patient group, red indicates genes representing the Alpha and Beta infection patient groups, and black represents genes representing the HC group. Each gene marked with an arrow on the Biplot is displayed as a heatmap, with values indicating log2 fold change of gene expression levels against HC. Hierarchical clustering was performed with a Euclidean distance matrix of fold changes.

To identify genes within the RNA splicing machinery that impact the abnormal regulation of alternative splicing in COVID-19 patients, we collected 304 genes related to RNA splicing genes with an FPKM exceeding 5 in at least one sample and conducted a PCA based on their expression values (Figure 4A). As a result, clear distinctions were made between HC and each patient group, while Alpha and Beta displayed similar trends. Moreover, a tendency of division based on vaccination status was observed within Omicron patients. This suggests that RNA splicing genes are regulated differently among patients infected with respective variants. Upon identifying the top 25 genes contributing significantly to the principal components distinguishing each group, it was observed that 11 genes contributing to HC were expressed at lower levels across all patient groups (Figure 4B). Additionally, five genes highly contributing to the Alpha and Beta groups exhibited group-specific elevated expression. Furthermore, nine genes in Omicron patients showed distinctively higher expression, with a notably high expression observed particularly in Omicron group 2. Through this, we deduce that the regulation of RNA splicing machinery genes may be associated with the variant-specific host responses to SARS-CoV-2 infection. The differential expression and regulatory patterns of these splicing machinery genes might play pivotal roles in the abnormal alternative splicing regulation observed in COVID-19 patients.

## Discussion

In this study, we probed the alternative splicing (AS) landscape in patients infected with various SARS-CoV-2 variants, aiming to illuminate potential underlying mechanisms contributing to the variance in disease manifestations and progression. Our exploratory analysis revealed a significant number of DASEs, notably in individuals infected with the Omicron variant. This observation necessitates an in-depth investigation into its broader impact on the host’s cellular and molecular responses. Particularly, the observed down-regulation of mRNA splicing machinery genes in COVID-19 patients and their variant-specific modulation signify the critical role of AS mechanisms in disease expression.

Recent studies have shown that infection by numerous viruses affects the AS landscape of host-cells.^40^^,^^41^^,^^42^^,^^43^^,^^44^^,^^45^^,^^46^^,^^47^^,^^48^ The modulatory activities of viral products on cellular AS, as reflected through the inhibition of host-cell splicing factor kinases^49^^,^^50^^,^^51^ and interference with splicing factors^52^^,^^53^ and spliceosome,^54^^,^^55^^,^^56^^,^^57^ manifest a complex battle between the virus attempting to subvert host defenses and the cell striving to counteract these invasions. The restoration of regular AS patterns may be used to stop viral spread and lessen the severity of the disease if these modulations are understood at a granular level, opening new treatment possibilities.

The analysis of 190 RNA-seq datasets, encompassing an average depth of 212 million reads, yielded a rich repository of deep sequencing data. This data depth accords a robust framework to navigate through the complex transcriptional landscape, enabling the detection of genes with low expression levels, and unraveling rare AS events. Building upon recent studies^13^^,^^15^^,^^16^^,^^55^^,^^58^ that highlighted the potential targeting of the mRNA splicing machinery by viruses and reported a dysregulation of AS in COVID-19 patients, our transcriptomic analysis including healthy controls and patients reinforced the global dynamic alterations of AS in COVID-19 patients. A notable extent of these changes was observed in those infected with the Omicron variant.

While we acknowledge the smaller number of RNA-seq samples in the Beta- and Gamma-infected patient groups, it is important to consider whether these small sample sizes could be contributing to the limited number of DASEs observed in these groups (Figure 1B). Interestingly, when comparing the number of samples between the Alpha-infected and Omicron-infected patient groups, we observed a similar sample size (45 vs. 46). However, despite this similarity, the count of DASEs in Alpha patients (2,245) did not substantially exceed that in Omicron patients. Moreover, we introduced multiple testing correction measures in the definition of DASEs, ensuring that the significance threshold for determining DASEs becomes more stringent with larger sample sizes. Despite these adjustments, our results suggest that the observed differences in DASE numbers between patient groups are not solely attributable to sample size variations.

Previous studies have underscored the up-regulation of *TLR4* in severe COVID-19 patients, implicating it in augmented *ACE2* expression and ensuing hyperinflammation.^59^^,^^60^ Among the AS forms of *TLR4*, the inclusion of exon 2 or exon 3 has been reported to be elevated in LPS treated cells, mirroring our observation of increased inclusion of these exons in COVID-19 patients.^29^^,^^30^ Extending the molecular narrative, transcriptome analyses on hospitalized patients infected with Alpha^21^ or Beta^20^ variants revealed a pronounced activation of interferon pathway genes with a spotlight on the Janus kinase (JAK)/signal transducer of activation (STAT) signaling pathway. For genes related to the JAK/STAT pathway, the alterations in their AS patterns during infection may have downstream consequences on the signaling cascade, potentially influencing immune responses and cytokine production.^61^ Concordantly, our analysis unearthed novel DASEs in *JAK* and *STAT* genes, hinting at a potential aberrant regulation of the JAK/STAT pathway in COVID-19 patients.^62^^,^^63^ However, the functional ramifications of these DASEs remain to be elucidated.

Wang et al. reported an increased exclusion form of exon 7 in *CD74* and *LRRFIP1* in the lung tissues of severe COVID-19 patients, alongside a significant down-regulation of six spliceosome component proteins.^15^ Our findings corroborated the increased exclusion of *CD74* exon 7 in patients, although we couldn’t confirm this for *LRRFIP1* (Figure S2). *CD74* is a gene that encodes a protein called the invariant chain (Ii), which plays a crucial role in antigen presentation. Antigen presentation is a process by which immune cells display fragments of foreign substances, known as antigens, on their surface to activate an immune response.^64^ Changes in the AS patterns of *CD74* during infection, such as in COVID-19 patients, may impact the presentation of antigens and subsequently influence immune responses. The AS of *CD74* can affect the processing and trafficking of major histocompatibility complex class II (MHC-II) molecules, which are responsible for presenting antigens to immune cells.^65^ Moreover, our findings point toward a general depression of splicing machinery genes, suggesting a complex interplay far beyond the direct interactions between viral proteins and spliceosome component proteins, potentially disrupting cellular functions and innate immunity.^12^^,^^41^^,^^42^^,^^43^ We also found that the variant- and vaccination-specific gene expression profiles of genes which are member of RNA splicing machinery, suggesting that global and variant specific regulation of AS is highly associated with transcriptional alterations of RNA binding proteins.^17^^,^^33^^,^^66^ This further suggests that the observed splicing gene depression might underlie the abnormal AS seen in COVID-19 patients.

Numerous genes, including those linked to innate immune activation and strong cytokine activity, have seen increased transcription levels in severe COVID-19 patients.^67^^,^^68^^,^^69^ This hyper-activation of transcription might encourage the production of cellular AS transcripts that were not intended.^70^ Our study reveals striking alterations in gene expression and AS profiles of numerous immune and cytokine-related genes in COVID-19 patients. Although we observed that AS rates are not correlated with transcription rates, functional studies are needed to know whether AS isoforms increased in patients are functional or transcriptional noise.

Omicron infections generally manifest more moderate symptoms compared to other variants,^71^ and vaccinated patients exhibit a significantly blunted interferon response compared to unvaccinated Omicron infected outpatients and unvaccinated Alpha infected hospitalized patients.^27^ On the gene expression, Omicron showed a gene expression profile more like healthy controls as compared to Alpha or Beta (Figure S1), yet AS exhibited a dynamic alteration, especially pronounced in the vaccinated group. Changes in AS following vaccination have been previously documented in the context of dengue virus vaccination.^72^ Our observations in AS changes of T and B cell receptor signaling pathway genes according to vaccination status hint at the possibility of altered immune cell populations or functionalities.

In our study, we have carefully considered the potential impact of prior infections on the interpretation of our data. It is worth noting that among the Omicron-infected patients, only 17.3% (8 out of 46) had known prior infection. However, patients were not screened for prior asymptomatic infection; therefore, we were unable to stratify or control for this factor in our analysis. We acknowledge this limitation in the interpretation of our results. Prior infections, akin to vaccination, can significantly impact the innate and adaptive immune response,^22^^,^^73^^,^^74^^,^^75^^,^^76^ potentially influencing AS patterns. Future studies with a larger cohort and more comprehensive patient information on symptomatic and asymptomatic infection status could provide a deeper understanding of how prior infections may impact AS in the context of SARS-CoV-2 infection.

We reported 25 members of RNA splicing machinery that are specifically expressed in each patient group. Noteworthy among these is the heterogeneous nuclear ribonucleoprotein A1 (HNRNPA1), known to interact with the 3′-UTR of viruses, orchestrating transcription and replication processes.^77^^,^^78^^,^^79^ Across all variant groups, *HNRNPA1* exhibited a common downregulation trend. Intriguingly, recent observations have spotlighted HNRNPA1 as a hub protein with substantial functional linkages to the human SARS-CoV-2 genome.^80^ Similarly, network pharmacology methods have accentuated a close nexus between COVID-19 and serine/arginine-rich splicing factor protein kinase-1 (SRPK1), a gene intimately involved in SARS-CoV-2 replication through phosphorylation of the N protein.^81^^,^^82^ Our analysis unveiled a pronounced expression of *SPRK1* predominantly in the Alpha and Beta infected groups, shedding light on possible variant-specific molecular dialogues orchestrated by the virus. We delved into the expression patterns of zinc finger CCCH-type containing 13 (ZC3H13), which has been reported to be closely associated with N^6^-methyladenosine (m^6^A), a prevalent epigenetic modification that regulates splicing efficiency^83^ and is found in the viral RNA genomes of various viruses.^84^ The function of m^6^A in these viral genomes has underscored the intricacies of host-virus interactions at the epigenetic level. One report in the literature found that *ZC3H13* is expressed at lower levels in COVID-19 infected individuals as compared to individuals with a non-COVID-19 infection.^85^ Our analysis unveiled that *ZC3H13* was specifically overexpressed in the Omicron-infected group, particularly in the vaccinated cohort, suggesting a possible interplay between epigenetic modifications and the host’s response to different SARS-CoV-2 variants post-vaccination. Through this exploration, we have laid down a significant marker, directing future research endeavors toward a deeper understanding of the host-virus interactions.

We analyzed gene expression levels of 20 immune cell markers from various patient groups to identify cell type heterogeneity in our buffy coat samples. Our results highlighted the diversity of immune cell populations found in the buffy coats by revealing different patterns of marker expression (Figure S3). Significant variations in the expression of markers linked to neutrophils and monocytes were noted in patients with Alpha and Beta infections.^21^ Omicron-infected patients, except for monocyte markers, showed marker expression patterns more in line with those of healthy controls. Moreover, it is worth noting that the average time interval between vaccination and infection in the vaccinated patients was approximately four months, providing sufficient time for any potential vaccine-induced changes in total white blood cell numbers or proportion of different cell types to subside. These results strengthen our main conclusion that the dramatic AS changes observed are primarily driven by Omicron infection and vaccination status, rather than variations in immune cell composition. While acknowledging the limitations related to the absence of direct flow cytometry data, our analysis of immune cell markers provides valuable insights into the relative composition of immune cell types within the buffy coats and supports our main findings.

We addressed the observations made concerning the PCA analysis of the inclusion level of AS events and the relatively small percentage of variation explained by the PC2 metric (Figures 1A and 3A). It is important to emphasize that PCA is a data reduction technique employed to visualize multidimensional data in a lower-dimensional space while retaining as much variability as possible. Therefore, while PC2 accounts for a smaller proportion of the total variance, it still captures meaningful differences in splicing patterns among the sample groups. To further validate our findings and consider alternative perspectives, we complemented the PCA analysis with a correlation-based approach. Specifically, we performed correlation analysis among samples based on the percent spliced in (PSI) values of AS events (Figure S4). This strategy allowed us to identify samples that exhibited high correlations in their splicing patterns and subsequently cluster them accordingly. Importantly, we observed that this clustering was influenced more by PC2 than PC1, which provides additional support for the relevance of PC2 in distinguishing between sample groups.

AS is mediated by interactions of *cis*-sequence elements and *trans-*acting splicing factors.^86^^,^^87^ To identify potential regulatory factors of DASEs in COVID-19 patients, we analyzed RNA binding protein motifs in 500 bp up and downstream sequences of cassette exons (Figure S5A). By analyzing enrichments of established human RNA binding motifs^88^ in the sequences we identified enriched motif clusters for each comparison group (HC vs. All patients, HC vs. Alpha, HC vs. Omicron, and Omicron group 1 vs. Omicron group 2) (Figure S5B; Table S2). These motifs shed light on potential RNA binding proteins involved in the regulation of AS events in the context of SARS-CoV-2 infection. Furthermore, we examined the gene expression profiles of these potential regulatory factors, showing dynamic expression in the COVID-19 patients (Figure S5C). These regulatory factors exhibited varying expression patterns across patient groups that may be associated with the DASEs observed in our analysis.

To determine whether alternative-spliced transcripts identified through our bioinformatics analyses could be experimentally validated, we chose three genes, *TLR4*, *LST1*, and *CLEC7A,* identified in Omicron-infected patients and randomly selected four Omicron-infected patient samples to test for expression of alternatively spliced isoforms. Within this group of four samples, we were able to validate each of the AS events although not every patient sample revealed the same AS patterns (Figure S6). This experiment provides representative examples of validation for these three genes. The experiment does not of course provide validation across all of the alternatively spliced genes within all 190 samples, but it does show that AS variants identified using bioinformatics can be experimentally validated at the wet bench. Our ability to perform thorough validation was limited by our inability to obtain cDNA samples from all the samples analyzed bioinformatically. As previously reported, *LST1* is a gene known for its extensive AS patterns and immunomodulatory function.^89^ *CLEC7A* has been linked to AS events during IAV infection.^90^ *TLR4* AS has been discussed earlier within this manuscript.^29^^,^^30^^,^^59^

In sum, our investigation unfurls a landscape elucidating the interplay between variant- and vaccination-specific transcriptional changes arising from alterations in gene expression and AS regulation in the context of SARS-CoV-2 infections. As we delved into the complex realm of AS, our analysis uncovered alterations that could significantly impact host immune responses, hinting at a critical layer of host-virus interaction that warrants further exploration. Through this comprehensive analysis, we aim to provide a robust framework for understanding how the interplay of viral genetic diversity, host transcriptomic modulation, and vaccination status contribute to the COVID-19 disease spectrum, thereby fostering a more informed foundation for future research and clinical interventions in COVID-19.

### Limitations of the study

There are several limitations to this study. First, our study encompassed a diverse set of patient groups, but sample size variations were evident, with smaller sample sizes in the Beta- and Gamma-infected groups. This discrepancy may have contributed to the limited number of observed DASEs in these groups. Second, while we made efforts to account for known prior infections in the Omicron-infected patients, our study did not screen for prior asymptomatic infections. Third, though we successfully identified DASEs associated with SARS-CoV-2 infection and vaccination status, the functional consequences of these events remain unclear. In-depth mechanistic insights into how these AS changes influence immune responses and disease outcomes will require further investigation. Fourth, our analysis of immune cell markers provided insights into the relative composition of immune cell types within the buffy coats. However, we did not directly quantify immune cell populations using flow cytometry, and there may be additional cell heterogeneity not captured in our analysis.

## STAR★Methods

### Key resources table

**Table:** 

REAGENT or RESOURCE	SOURCE	IDENTIFIER
**Deposited data**		

RNA-seq data (57 Healthy controls)	Lee et al.^26^	GSE162562
RNA-seq data (14 Healthy controls)	Lee et al.^25^	GSE190747
RNA-seq data (8 Healthy controls and 9 Beta infected patients)	Knabl et al.^20^	GSE189039
RNA-seq data (45 Alpha infected patients and 3 Gamma infected patients)	Lee et al.^21^	GSE190680
RNA-seq data (8 Healthy controls and 46 Omicron infected patients)	Lee et al.^27^	GSE201530

**Critical commercial assays**		

DreamTaq Green PCR Master Mix	ThermoFisher Scientific	Cat# K1081

**Oligonucleotides**		

Primers for PCR, see Figure S6	Eurofins Genomics	

**Software and algorithms**		

SRA toolkit (v.3.0.0)	Leinonen et al.^91^	https://github.com/ncbi/sra-tools
HISAT2 (v.2.2.1)	Kim et al.^92^	http://daehwankimlab.github.io/hisat2
rMATs (v.3.2.5)	Shen et al.^28^	https://rnaseq-mats.sourceforge.io
Cufflinks (v.2.2.1)	Roberts et al.^93^	https://github.com/cole-trapnell-lab/cufflinks
DAVID (v.6.8)	Dennis et al.^94^	https://david.ncifcrf.gov
ShinyGO (v.0.77)	Ge et al.^95^	http://bioinformatics.sdstate.edu/go
STRING (v.12.0)	Szklarczyk et al.^96^	https://string-db.org
R (v 4.3.1)	https://www.r-project.org	NA

### Resource availability

#### Lead contact

Further information and requests for resources should be directed to the lead contact, Hye Kyung Lee (hyekyung.lee@nih.gov).

#### Materials availability

This study did not generate new unique reagents.

#### Data and code availability

• Differentially alternative spliced events (DASEs) data from this study is available on our GitHub repository: https://github.com/tjdrnjsqpf/COVID19_AS.
• This paper does not report original code.
• Any additional information required to reanalyze the data reported in this paper is available from the lead contact upon request.

### Method details

#### Data collection

We obtained whole blood RNA-seq data from COVID-19 patients infected with four different variants (Alpha, Beta, Gamma, and Omicron), as well as from a group of healthy controls, using previously published data from five Gene Expression Omnibus (GEO) datasets (see Table S1 for details).^20^^,^^21^^,^^22^^,^^25^^,^^26^^,^^27^ The healthy control group comprised 87 samples, while the COVID-19 patient group consisted of 103 samples. To focus on early-stage infection, we exclusively collected data from patients who had been infected for less than one week. All raw sequences were downloaded and converted to FASTQ by SRA toolkit (v.3.0.0).^91^

#### Alternative splicing and transcriptome analyses

The raw reads were initially subjected to preprocessing, involving the removal of poor-quality 3’ ends, utilizing the *trimFastq.py* script, which is an integral component of rMATs (v.3.2.5).^28^ Subsequently, the resulting cleaned reads were then mapped to the human genome using HISAT2 (v.2.2.1)^92^ with default parameter settings. Furthermore, an HISAT2 genome index was constructed as part of the preparation for this mapping step. To identify alternative splicing events, we ran rMATs with default parameters and human gene annotations. We utilized a widely recognized exon-based ratio metric known as the percent spliced in index (PSI) ratio to quantify alternative splicing events. The PSI ratio is calculated as follows:

$$PSI=\frac{I/L_{I}}{{S/L}_{S}+{I/L}_{I}}$$

where *S* and *I* represents the number of reads mapped to the junction supporting the skipping and inclusion form, respectively. *L* signifies the effective length, which is used for normalization.

To identify differential alternative splicing events (DASEs), we conducted a comparison of exon inclusion levels between healthy control and COVID-19 patients for each alternative splicing event. DASEs were defined using the following criteria: |PSI differences| > 0.1 and corrected *P*-value < 0.05. For the statistical analysis, we employed the Wilcoxon rank-sum test, and obtained corrected *p*-value by Bonferroni correction.

To quantify gene expression levels, we employed Cufflinks (v.2.2.1)^93^ to assemble genome-aligned reads into transcripts. The relative abundances of these transcripts were estimated based on the read count support for each transcript. Unless otherwise stated, we utilized fragments per kilobase per million reads mapped (FPKM) as the unit of gene expression levels. Differentially expressed genes (DEGs) were defined as genes with a significant expression difference between groups, characterized by a corrected p-value lower than 0.05 in group-to-group comparisons. The human reference genome sequence and annotation files were acquired from the UCSC genome browser (https://genome.ucsc.edu) under version hg38. Additionally, we excluded uncharacterized and alternative chromosomes and their associated genes from our analysis.

#### Gene set enrichment and over-representation analysis

We performed over-representation analysis for pathway enrichment of DASEs and DEGs using DAVID (v.6.8)^94^ (https://david.ncifcrf.gov) and ShinyGO (v.0.77)^95^ (http://bioinformatics.sdstate.edu/go). In this analysis, we selected significant categories and pathways with a false discovery rate (FDR) less than 0.05 for further investigation. The gene set of gene ontology (GO) terms and pathways were obtained Gene Ontology resources (https://geneontology.org) and Kyoto Encyclopedia of Genes and Genomes (KEGG) pathway database (https://www.genome.jp/kegg/pathway).

#### SARS-CoV-2 susceptible genes

SARS-CoV-2 susceptible genes were collected form PanelApp,^97^ a resource that provides curated information about genes and their associations with specific diseases and conditions, specifically utilizing the panel of COVID-19 research (v.1.136) (https://panelapp.genomicsengland.co.uk/panels/111).

#### Protein-protein interaction (PPI) network analysis

We construct a PPI network from DEGs of COVID-19 patients, specifically enriched in RNA splicing term (GO:0008380) by STRING database (v.12.0)^96^ (https://string-db.org). We employed stringent parameters, requiring interactions with the highest confidence (minimum required interaction score > 0.9) and hiding disconnected nodes in the network.

#### Motif analysis

The 500 bp up- and downstream of sequences of cassette exons which differentially included between four major comparisons (HC vs. All patients, HC vs. Alpha, HC vs. Omicron, and Omicron group 1 vs. Omicron group 2) were extracted. The XSTREME package^98^ was employed to identify enriched binding motifs of RNA binding proteins in the XSTREME database (Ray 2013 human). By default, XSTREME reports 6- to 15-mer motifs whose E-value < 0.05. The program uses Fisher’s exact test or the binomial test to determine the significance of each motif found.

#### PCR of alternative splicing variants

cDNA samples used this study were previously published^22^ and PCR was performed with DreamTaq Green PCR Master Mix (Thermo Scientific, Gaithersburg, MD, USA) under the following conditions: denaturation at 98°C for 3 min; 40 cycles of 98°C for 30 s, 58°C for 30s, and 72°C for 90 s, and final extension at 72°C for 10 min. The PCR products were visualized by electrophoresis through a 2% agarose gel. The PCR primers for *TLR4*, *LST1*, and *CLEC7A* of splicing variants are shown in Figure S6A.

### Quantification and statistical analysis

#### Data analysis

Principal component analysis (PCA) was performed using the exon inclusion levels of all alternative splicing events by *prcomp* function from R *stat* package with the first two principal components. To cluster the samples based on alternative splicing profiles, we conducted *k*-means clustering, which is one of the unsupervised clustering methods. To determine the optimal number of clusters (*k*), we performed multiple analyses by setting k from 2 to 5. Through this iterative process, we examined the saturation points of the total within sum of squares (SS) values and identified that the optimal value for *k* is 2. To assess the degree of gene contributions to principal components, we utilized *biplots* from the R *stat* package. The *p* values from comparing distributions were obtained by Wilcoxon rank-sum test. All *p* values were adjusted by Bonferroni correction.

## Supplemental information

Document S1. Figures S1–S6

Table S1. RNA-seq data statistics, related to Figure 1

Table S2. Motif analysis of DASEs, related to Figures 3 and S5
