# Supplementary material for: Variant- and vaccination-specific alternative splicing profiles in SARS-CoV-2 infections
Source: iScience. 2024 Feb 8;27(3):109177. doi: 10.1016/j.isci.2024.109177 (PMC10897911; doi:10.1016/j.isci.2024.109177)
Supplement: Document S1. Figures S1–S6 [file mmc1.pdf]

iScience, Volume *27*

Supplemental information

Variant- and vaccination-specific alternative splicing profiles in SARS-CoV-2 infections

Sung-Gwon Lee, Priscilla A. Furth, Lothar Hennighausen, and Hye Kyung Lee

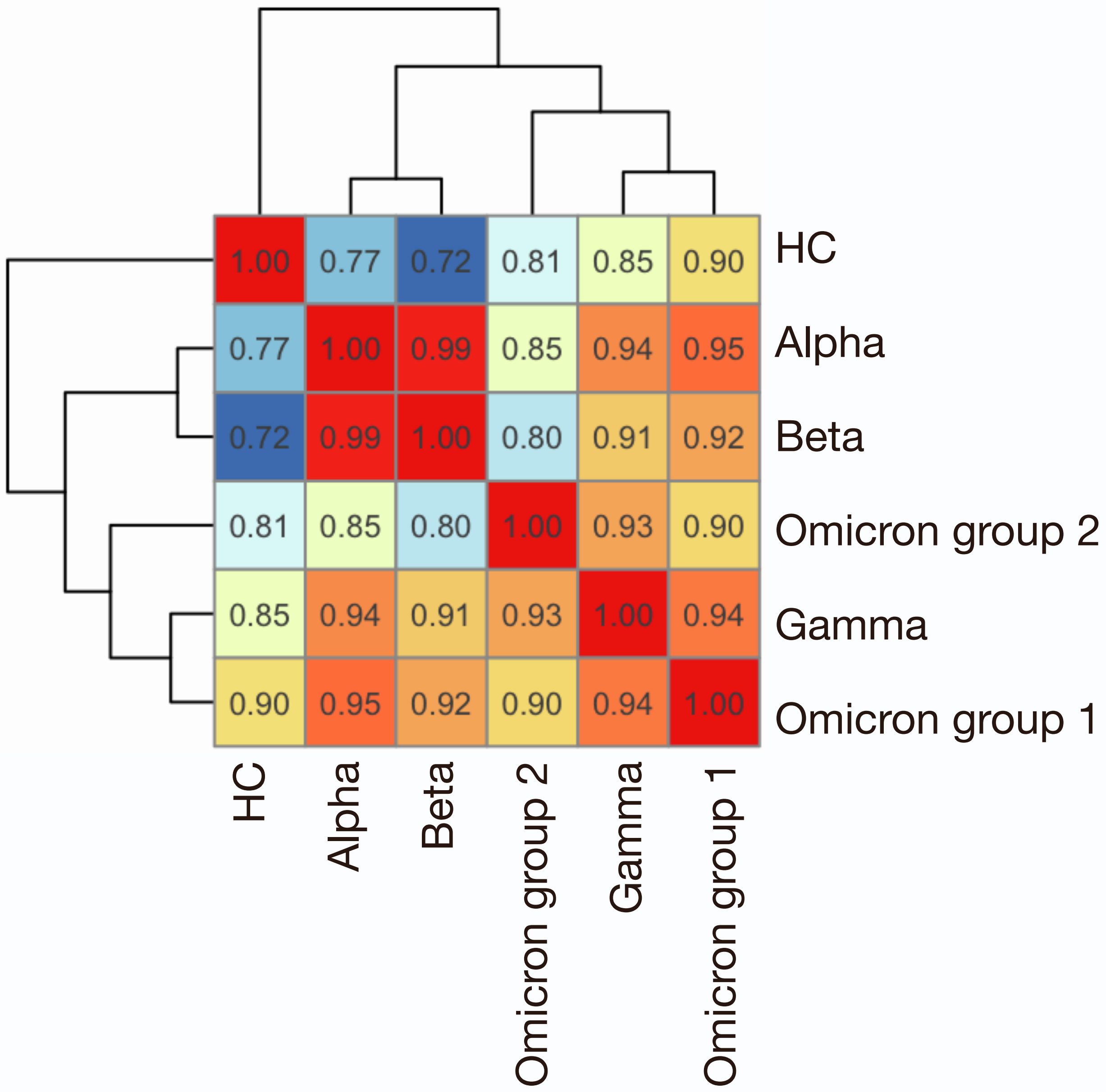

Supplementary figure 1. Gene expression level correlation between groups, related to Figure 1. Each value indicates Pearson’s correlation coefficient. Hierarchical clustering was performed based on Euclidean distance matrix.

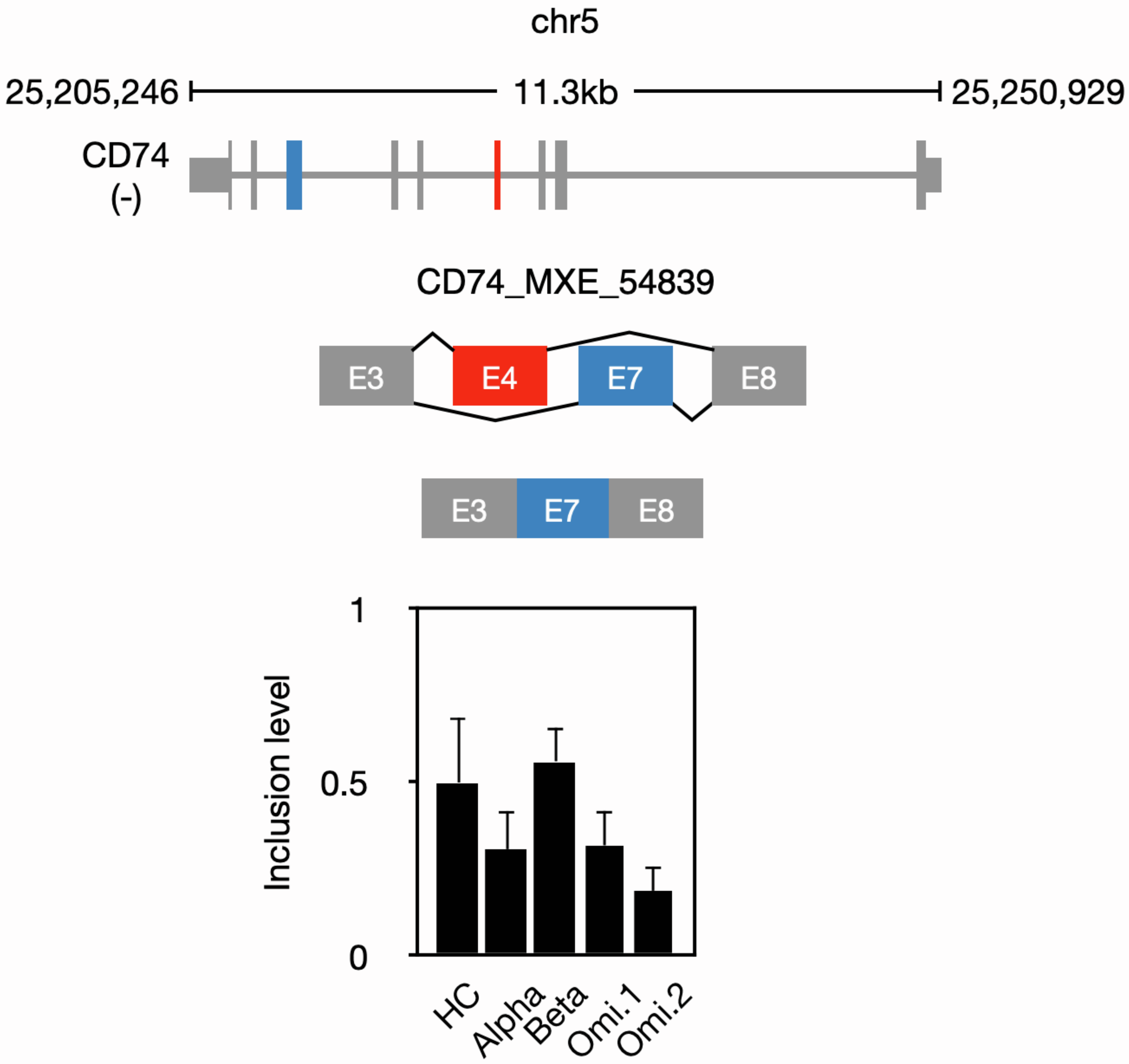

Supplementary figure 2. DASEs of *CD74* of which exon 7 significantly excluded in COVID-19 patients, related to Figure 3. The error bar indicates standard deviation of the mean.

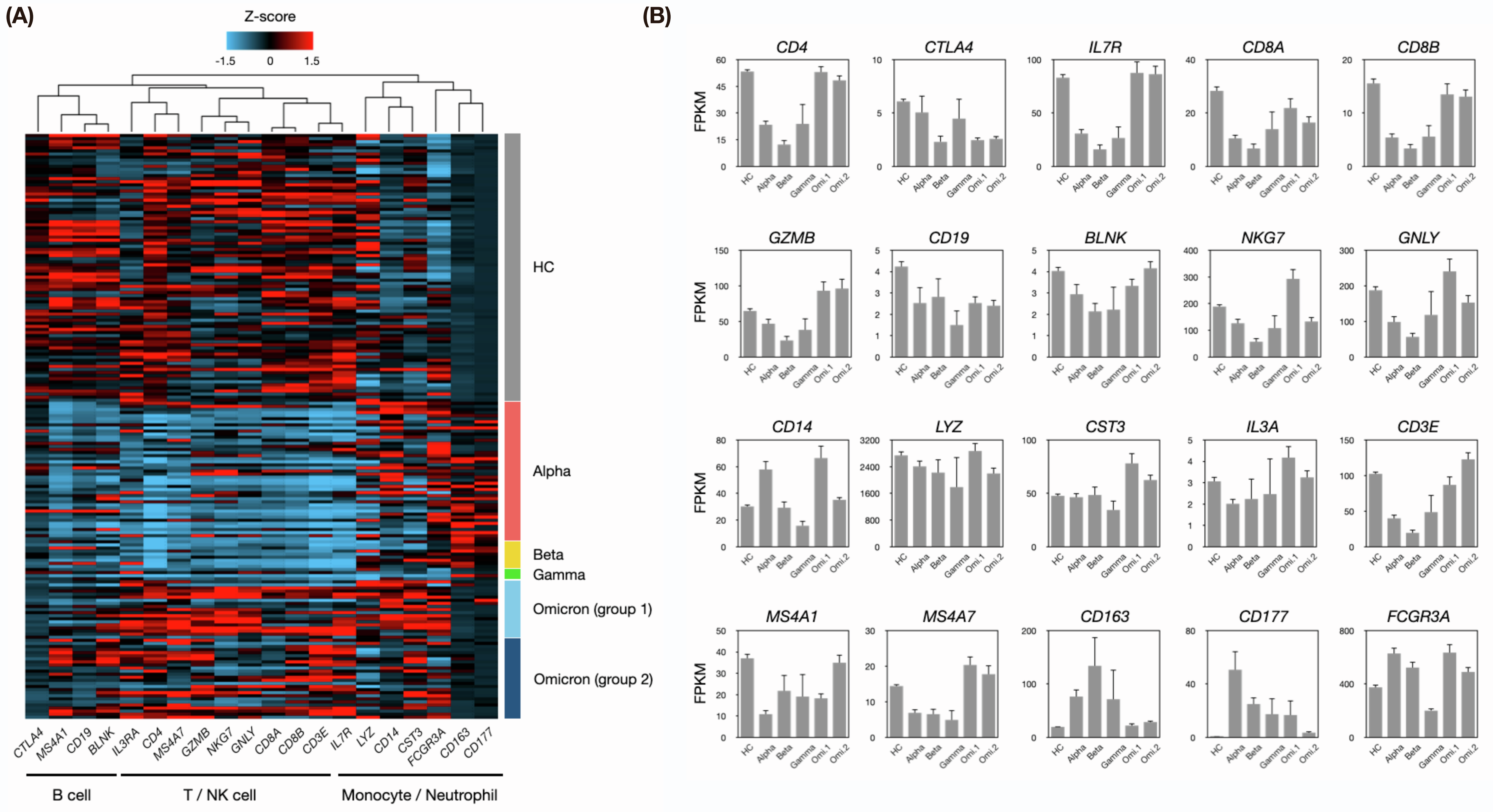

Supplementary figure 3. Gene expression levels of 20 immune cell markers, related to Figure 1. (A) Relative gene expression levels and **(B)** absolute FPKM of 20 immune cell markers. Hierarchical clustering of DEGs was performed with Euclidean distance matrix of relative gene expression levels. The error bar indicates standard error of the mean.

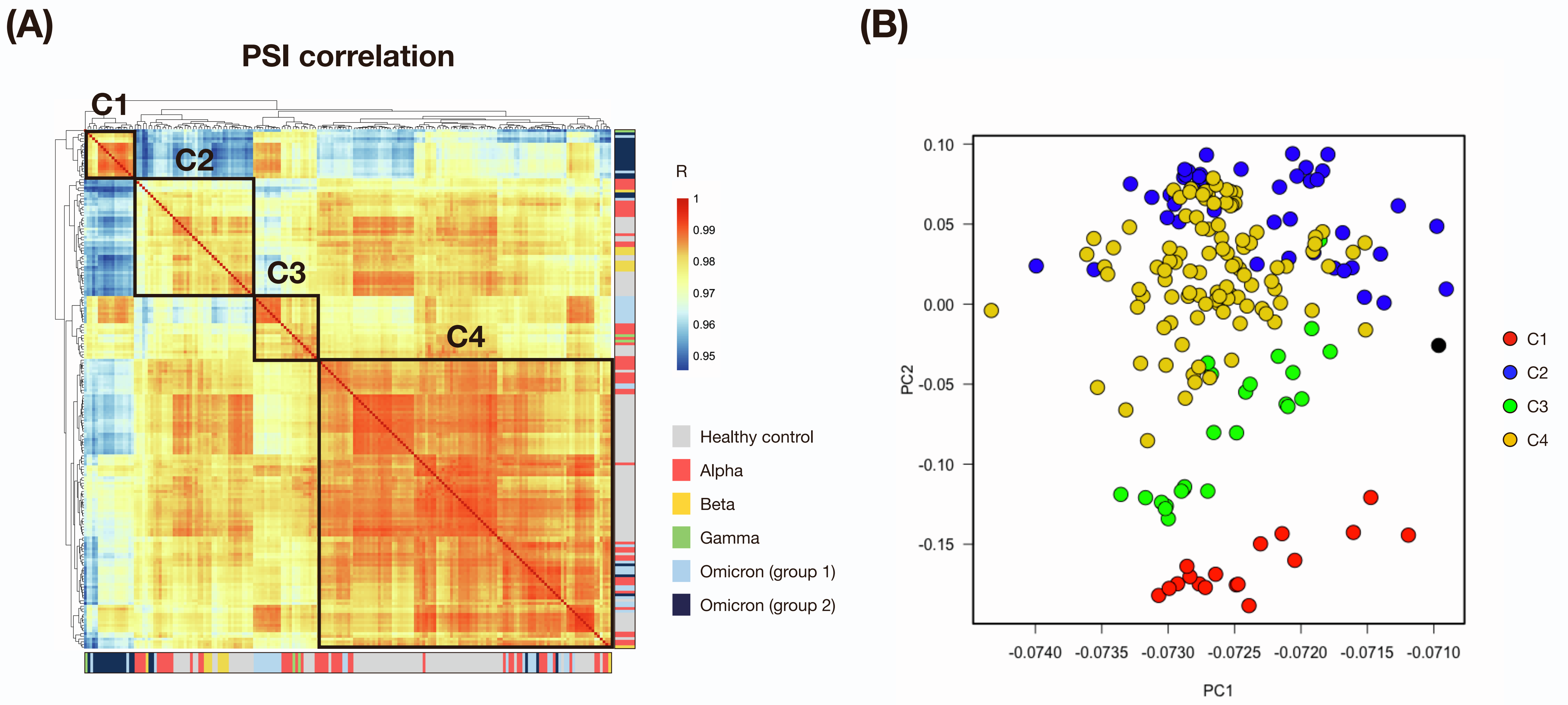

Supplementary figure 4. Clustering via alternative splicing profiles, related to Figure 1. (A) Correlation coefficient matrix of percent spliced in (PSI) values across samples. **(B)** Samples of original PCA plot were marked according to clusters of correlation analysis.

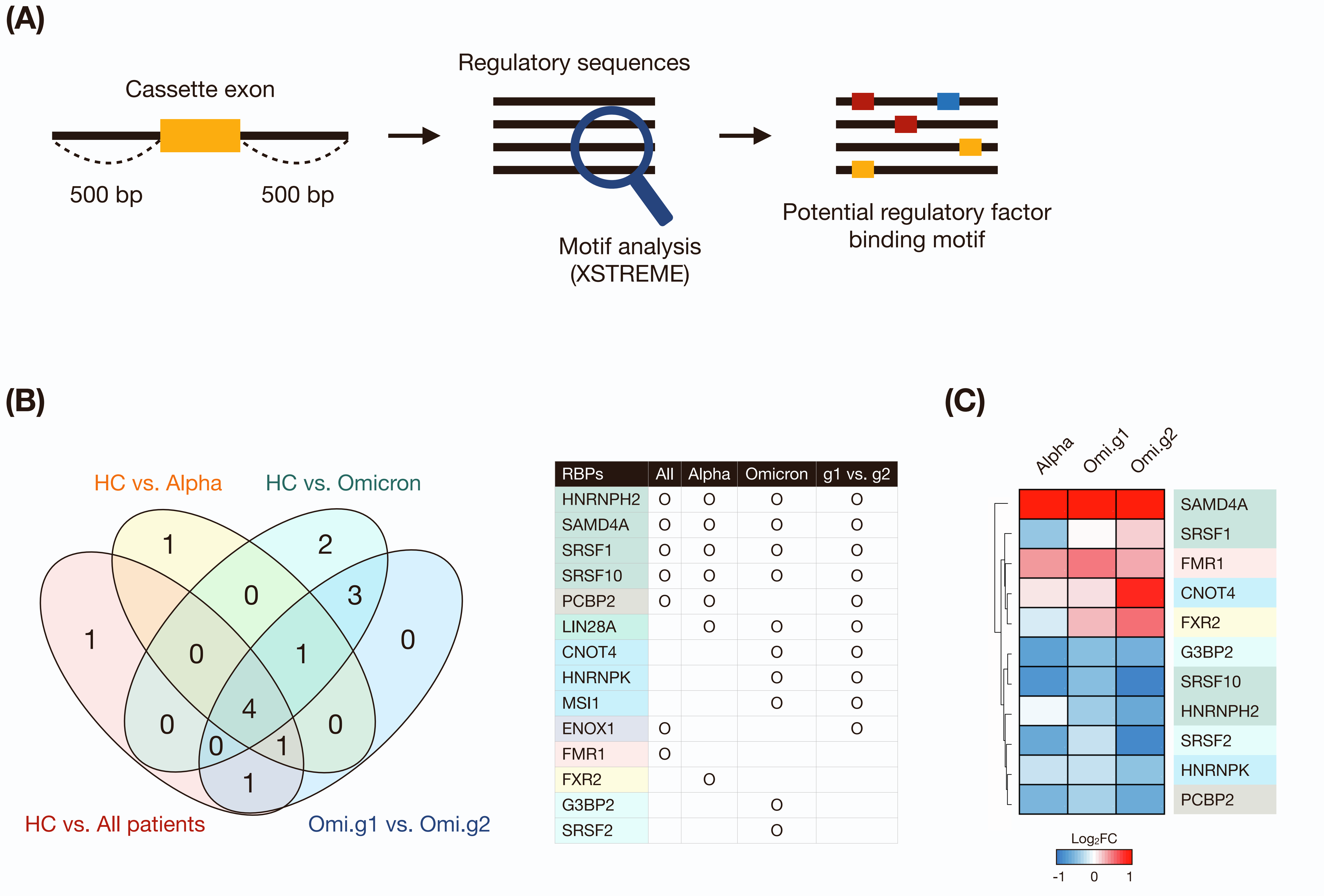

RBPs	All	Alpha	Omicron	g1 vs. g2
HNRNPH2	O	O	O	O
SAMD4A	O	O	O	O
SRSF1	O	O	O	O
SRSF10	O	O	O	O
PCBP2	O	O		O
LIN28A		O	O	O
CNOT4			O	O
HNRNPK			O	O
MSI1			O	O
ENOX1	O			O
FMR1	O			
FXR2		O		
G3BP2			O	
SRSF2			O	

Supplementary figure 5. RNA binding protein motif analysis within alternative spliced genes, related to Figure 4. (A) A schematic diagram of motif analysis. We extracted ± 500bp sequences of cassette exon that alternatively included or excluded in DASEs and analyzed enriched RNA binding protein motif (Ray2013 Homo sapiens) using the XSTREME package. **(B)** RNA binding proteins from Top 5 enriched motif clusters of each comparison group (HC vs. All patients, HC vs. Alpha, HC vs. Omicron, and Omicron group 1 vs. Omicron group 2). **(C)** Gene expression profiles of potential DASEs regulatory factors. The heatmap was depicted by the average gene expression levels of log_2 fold change to HC. *ENOX1*, *LIN28A* and *MSI1* were excluded because their expression level was FPKM < 1 in all samples.

(A)

Target gene	Primer sequence (5'-3')
TLR4	F: ATGATGCCAGGATGATGTCTGCC
	R:AGAGGTGGCTTAGGCTCTGATATG
LST1	F: TCACCAGCCCCTGATCATTTC
	R: AGCAATGCAGGCATAGTCAGC
CLEC7A	F: ACAGACAGTCATCTCAGGAGCAG
	R: AGGAGATTAGAGCCCAGTTGCC

(B)

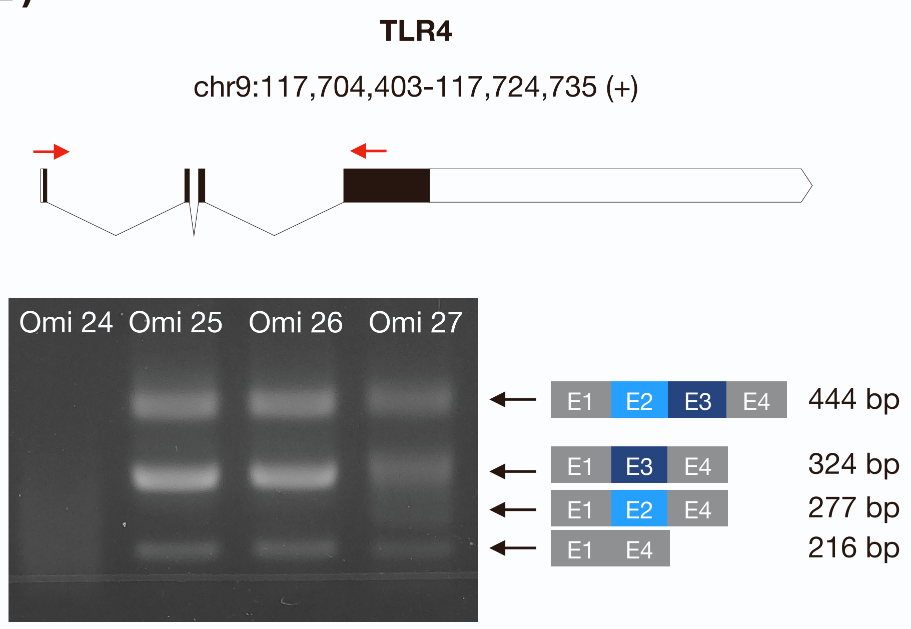

(C)

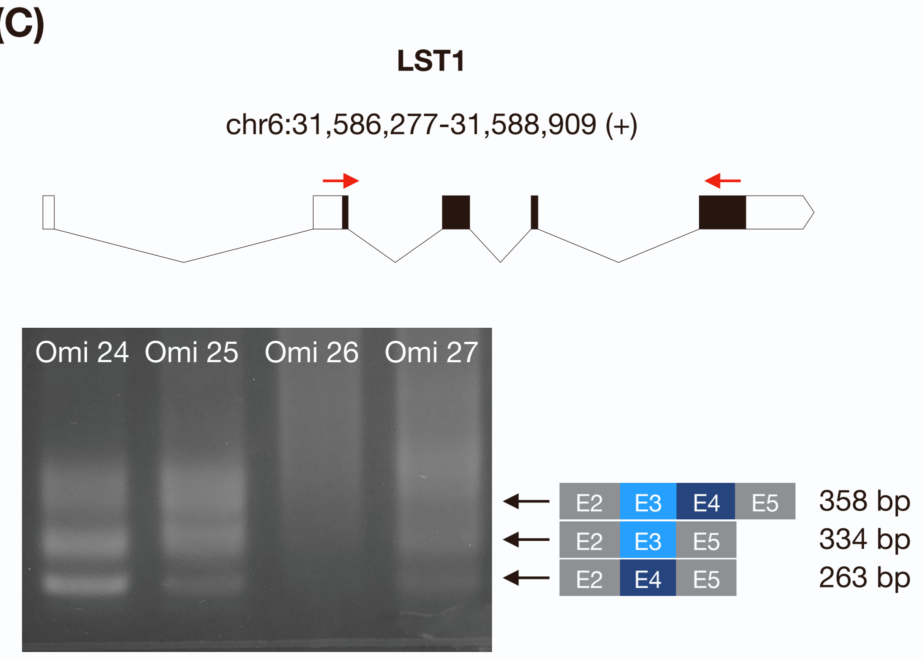

(D)

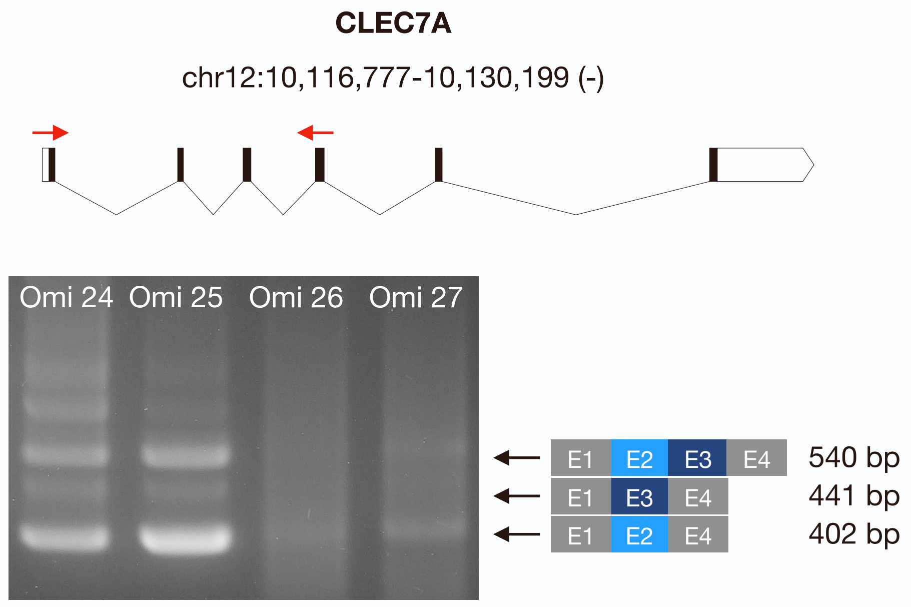

Supplementary figure 6. Experimental validation of expression of alternative spliced variants in Omicron infected patients, related to Figure 3. (A) PCR primers designed for three genes. Using these primers, we conducted **(B)** *TLR4*, **(C)** *LST1*, and **(D)** *CELC7A* PCR experiments, confirming the presence of each respective isoform. The red arrows indicate the primer positions. PCR was performed with DreamTaq Green PCR Master Mix (Thermo Scientific, Gaithersburg, MD, USA) under the following conditions: denaturation at 98°C for 3 min; 40 cycles of 98°C for 30 s, 58°C for 30s, and 72°C for 90 s, and final extension at 72°C for 10 min. The PCR products were visualized by electrophoresis through a 2% agarose gel.
